# Atomic-Scale Degradation Mechanisms during Nanoparticle Exsolution in Thin Films

**DOI:** 10.1021/acsnano.5c20606

**Published:** 2026-04-15

**Authors:** Yaolong Xing, Hyojin Yoon, Haeseong Jeong, Dongchang Kim, Stuart S. P. Parkin, Hyeon Han, Sang Ho Oh

**Affiliations:** † Institute for Energy Materials and Devices, 611524 Korea Institute of Energy Technology (KENTECH), Naju 58330, Republic of Korea; ‡ Department Structure and Nano-/Micromechanics of Materials, Max-Planck-Institute for Sustainable Materials, Dusseldorf 40237, Germany; § Department of Materials Science and Engineering, Pohang University of Science and Technology (POSTECH), Pohang 37673, Republic of Korea; ∥ Korea-Max Planck-PSI Center for Quantum Emergent Spintronics (KOMQUEST), Pohang 37673, Republic of Korea; ⊥ Max Planck Institute of Microstructure Physics, Weinberg 2, Halle (Saale) 06120, Germany; # Department of Energy Engineering, Korea Institute of Energy Technology (KENTECH), Naju 58330, Republic of Korea

**Keywords:** exsolution, nanoparticles, thin films, phase transformations, transmission electron microscopy

## Abstract

Exsolution, defined as the emergence of nanoparticles from a host matrix under reducing conditions, has become a key strategy in catalysis and energy applications, owing to its exceptional particle dispersion and stability. Although extensive research has focused on the early stage of exsolution, the atomic-scale degradation processes during prolonged nanoparticle exsolution remain poorly understood. Here, using in situ transmission electron microscopy, we directly visualize the sequential transformation and degradation processes during exsolution in nonstoichiometric La_0.2_Sr_0.7_Ni_0.1_Ti_0.9_O_3‑δ_ thin films. At the early stage, Ni nanoparticles were exsolved through a two-step crystallization process mediated by surface segregation. Remarkably, the nonstoichiometric perovskite lattice undergoes the annihilation of antiphase boundaries, known as fast diffusion channels for exsolution, through a transition toward a stoichiometric state induced by exsolution of Ni from the lattice. Upon continued high-temperature reduction, the film undergoes surface pit formation and progressive sublimation of Ni and Sr, accompanied by the emergence of a secondary La_2_TiO_5_ phase via surface ledge migration. These sequential transformations and degradation processes, intimately coupled to nanoparticle stability, provide mechanistic insight into the dynamic nature of exsolution and establish guiding principles for the design of catalytic and energy materials.

## Graphical abstract

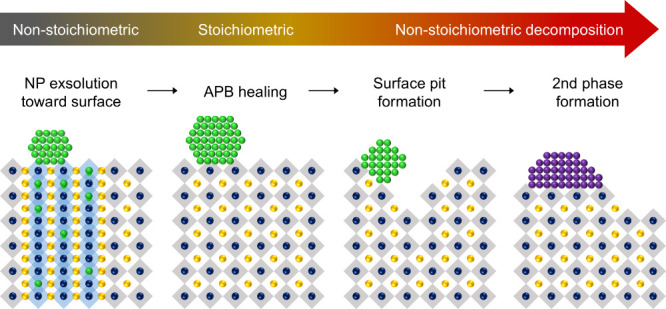

## Introduction

The controlled emergence of nanoparticles (NPs) through exsolution from a supporting matrix holds significant promise for catalysis and energy storage/conversion.^1–34^ In contrast to conventional NP deposition methods, exsolution allows for a uniform dispersion of NPs with a narrow size distribution.^2,5,6^ Additionally, the resulting socketed NP structure imparts outstanding thermal stability and resistance to coking, attributed to the robust interaction between the anchored particles and the oxide support.^3,21^ ABO_3_ perovskites, recognized for their versatility in composition, remarkable structural diversity, tunable properties, thermal stability, and proven applicability, offer an enticing platform for delving into the exsolution process.^1,5,22–24^ Particularly, nonstoichiometric perovskite oxides with A-site deficiency have attracted great attention because they acts as a general driving force for the enhanced exsolution of B-site cations into NPs while restoring the ABO_3_ stoichiometry upon reduction.^2,6^

Although the exsolved NPs can exhibit improved thermal stability due to the embedded nanostructure, the performance deterioration in energy conversion devices^25,26^ and the formation of a secondary phase after the prolonged exsolution^27^ have been reported. Therefore, understanding the coarsening and degradation mechanisms during exsolution, particularly at the atomic scale, is crucial for improving the long-term stability and performance of exsolution-based devices. Epitaxial thin films serve as ideal model systems for such investigations, because their well-defined crystal structures allow direct tracking of structural and morphological evolution during exsolution. Recently, plan-view imaging experiments have revealed key exsolution phenomena, including lateral migration and coalescence of exsolved NPs.^28–30^ Furthermore, it has been reported that charged perovskite surfaces significantly influence the surface mobility and thermal stability of NPs.^30^ Cross-sectional imaging of epitaxial thin films has further provided insights into particle nucleation and subsequent migration toward the surface.^31^ These studies have demonstrated the critical role of extended defects, such as antiphase boundaries (APBs)^31^ and dislocation cores,^28^ which act as preferential NP nucleation sites and migration pathways. Although extensive efforts have been made to understand the exsolution dynamics and coarsening mechanisms, most studies have primarily focused on the early stages of exsolution and therefore have not yet provided a comprehensive understanding of the coarsening and degradation processes, particularly at elevated temperatures.

Here, we employ in situ scanning transmission electron microscopy (STEM) in conjunction with electron energy-loss spectroscopy (EELS) to delve into the transformation and degradation mechanisms of the exsolved NPs in thin films of La_0.2_Sr_0.7_Ni_0.1_Ti_0.9_O_3‑δ_ (LSNT) with 10% A-site cation deficiency. We reveal a two-step crystallization process via surface segregation in the early-stage exsolution of Ni. The oxygen-deficient APBs, recognized as rapid diffusion pathways for exsolution,^31^ are annealed out upon temporarily restoring the ABO_3_ stoichiometry by the exsolution of Ni particles. Further reduction exhibits the formation of pits on the film surface, and the higher temperature reduction above ∼900 °C leads to the sublimation of Ni and Sr, thereby inducing the formation of a secondary phase, La_2_TiO_5_, through surface ledge migration. Thus, the two-step chemical state changes, i.e., nonstoichiometric to stoichiometric to nonstoichiometric transitions, are observed at prolonged reduction. Consequently, these findings uncover a fundamental relationship among lattice chemistry, NP stability, and sequential phase evolution, providing comprehensive insights for the design of advanced catalysts and energy devices.

## Results and Discussion

### Ex Situ Characterizations of Pristine and Exsolved Thin Films

60 nm-thick LSNT (001) thin films were grown on GdScO_3_ (GSO) (110) substrates with a misfit strain of +1.2% using pulsed laser deposition (PLD). The grown films were reduced in a tube furnace with a continuous flow of a gas mixture of Ar (95%) and H_2_ (5%) at 850 °C for 12 h and 950 °C for 2 h, respectively. Theta–2theta X-ray diffraction (XRD) (Figure 1a) shows the shift of the pristine film peak position to a higher angle after reduction at 850 °C, indicating contraction of out-of-plane (OOP) lattice parameter from 3.99 to 3.87 Å. This is due to a transition from nonstoichiometric to stoichiometric lattice after B-cation exsolution.^2,6^ However, it is noticeable that the OOP lattice parameter of the 950 °C reduced film (3.89 Å) is slightly increased compared to that of the 850 °C reduced film (3.87 Å), indicating further changes in the chemical state of the perovskite lattice, i.e., the formation of defects under the severe reduction environment. The sequential evolution of the chemical state was confirmed by in situ EELS measurements, as discussed later.

**Figure 1:**
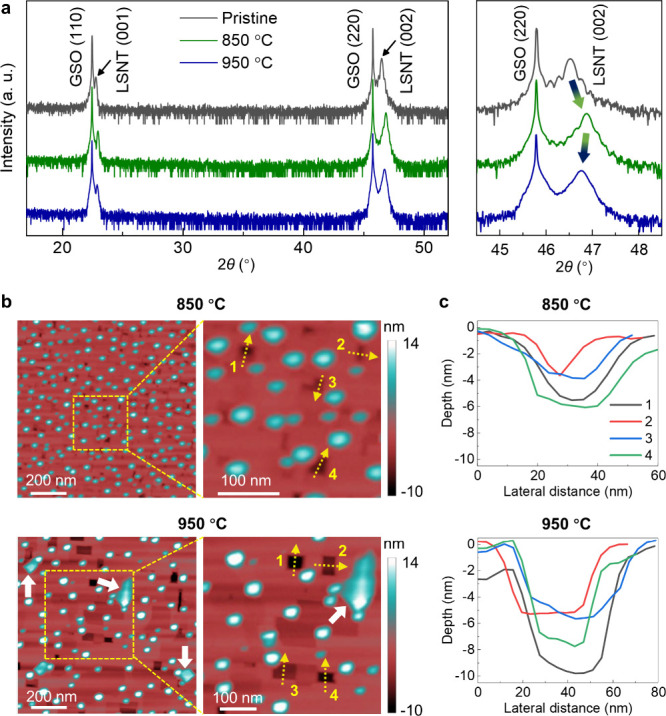
Epitaxial thin film growth and reduction. (a) Theta–2theta scans of pristine and reduced LSNT (001) films grown on GSO (110) substrates. The left and right panels represent wide and narrow range scans, respectively. (b) AFM maps of pristine and reduced films. The distinct secondary phases (marked by white arrows) are formed after reduction at 950 C. The right panels indicate the magnified AFM images of the 850 and 950 °C reduced films. The dashed lines indicate the regions where depth measurements were taken. (c) Depth profiles of pits on each film. The reduction was performed in a tube furnace with a continuous flow of a gas mixture of Ar (95%) and H_2_ (5%) at 850 °C for 12 h and 950 °C for 2 h, respectively.

Atomic force microscopy (AFM) measurements (Figure 1b,c and Figure S1) were conducted to examine the changes in surface topography under different reducing conditions. The pristine film exhibits a smooth surface with a root-mean-square (RMS) roughness of 0.22 nm (Figure S1). In contrast, after reduction, the film shows a significant increase in surface roughness, with RMS values of 3.15 and 3.96 nm after reduction at 850 and 950 °C, respectively. The pristine film does not show NP formation on the surface, as further confirmed by the cross-sectional TEM measurement (Figure 2), whereas the reduced film exhibits the formation of densely populated NPs on the surface. The density of NPs decreases from 212 to 80 particles·μm^–2^, while their average size increases from 10.7 ± 2.0 to 14.1 ± 3.4 nm for the films reduced at 850 and 950 °C, respectively. This corresponds to an approximately 15% decrease in the total volume of the surface NPs within the same area for the 950 °C reduced film compared to the 850 °C reduced one, suggesting volatilization of exsolved Ni NPs during the reduction at the higher temperature. Moreover, larger particles (indicated by white arrows) distinct from Ni NPs are observed on the 950 °C reduced film surface, implying the formation of a secondary phase at the elevated temperature.

**Figure 2:**
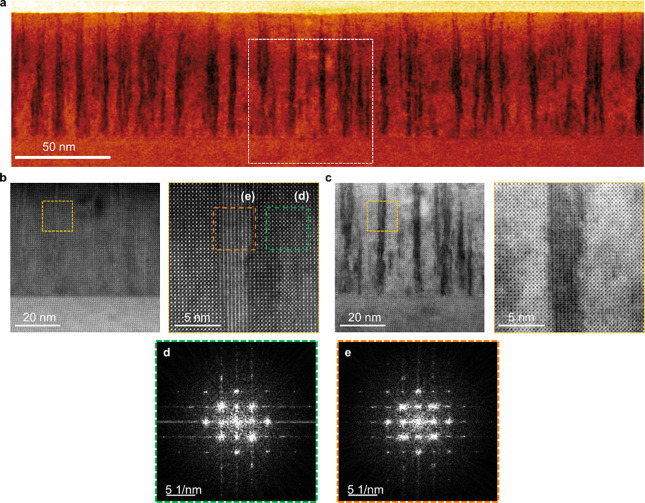
APBs in pristine thin films. (a) Low-magnification ABF-STEM image, highlighting the presence of APBs. The blocked region is shown in high magnification in panels (b) and (c), displaying the microstructure of APBs in detail through HAADF- and ABF-STEM imaging, respectively.

X-ray photoelectron spectroscopy (XPS) was employed to analyze the chemical states of Ni and Ti in the pristine and exsolved thin films (Figure S2). The pristine film mainly exhibits Ni^2+^ components, indicating that Ni species are primarily incorporated within the perovskite lattice. After exsolution, the XPS spectra show a clear increase in the metallic Ni^0^ peaks, accompanied by a decrease in Ni^2+^ intensity. Quantitative analysis of the fitted peak area ratios reveals that metallic Ni^0^ accounts for 70–80% of the total Ni content in the reduced films, indicating the formation of exsolved Ni NPs on the surface. In addition, the Ti^3+^ fraction increases from about 10% in the pristine film to 22 and 26% in the 850 and 950 °C reduced films, respectively, suggesting progressive reduction of Ti during the reduction process.

### APB Healing during Exsolution

Cross-sectional STEM imaging of the pristine film shows extensive arrays of vertical APBs, whose spatial extension appears as 2D projections of inclined APBs with widths ranging from 3 to 4 nm (Figure 2), consistent with previous observations in nonstoichiometric perovskite thin films.^31^ Local FFT analysis of the APB regions shows no additional reflections compared with the bulk, confirming the structural integrity of the APB, as its formation does not alter the crystal symmetry in the projected view.

Figure 3a and Movies S1 and S2 show the evolution of APBs during in situ STEM reduction experiments. At 700 °C, a significant fraction of APBs disappears, accompanied by the formation of Ni NPs on the film surface. Upon further annealing at 900 °C, pronounced ripening between NPs occurs while the APBs are completely removed. To elucidate the atomistic mechanism underlying the extinction of APBs, we scrutinized the motion of APBs in detail. Figure 3b describes the behavior of APBs observed along the ⟨110> zone axis. Heavy A-site cations (La/Sr) (bright atoms), outlined by dashed lines, from the antiphase nanodomain (APND) overlie the B-site (Ni/Ti) (less bright atoms) position of the matrix, resulting in complex intensity patterns in the APB region. At 200 s, the A-site sublattice of APND displaces from its initial position to halfway toward the A-site position of the matrix. By 344 s, A-site atoms from both APND and the matrix completely overlap, leading to annihilation of the APBs.

**Figure 3:**
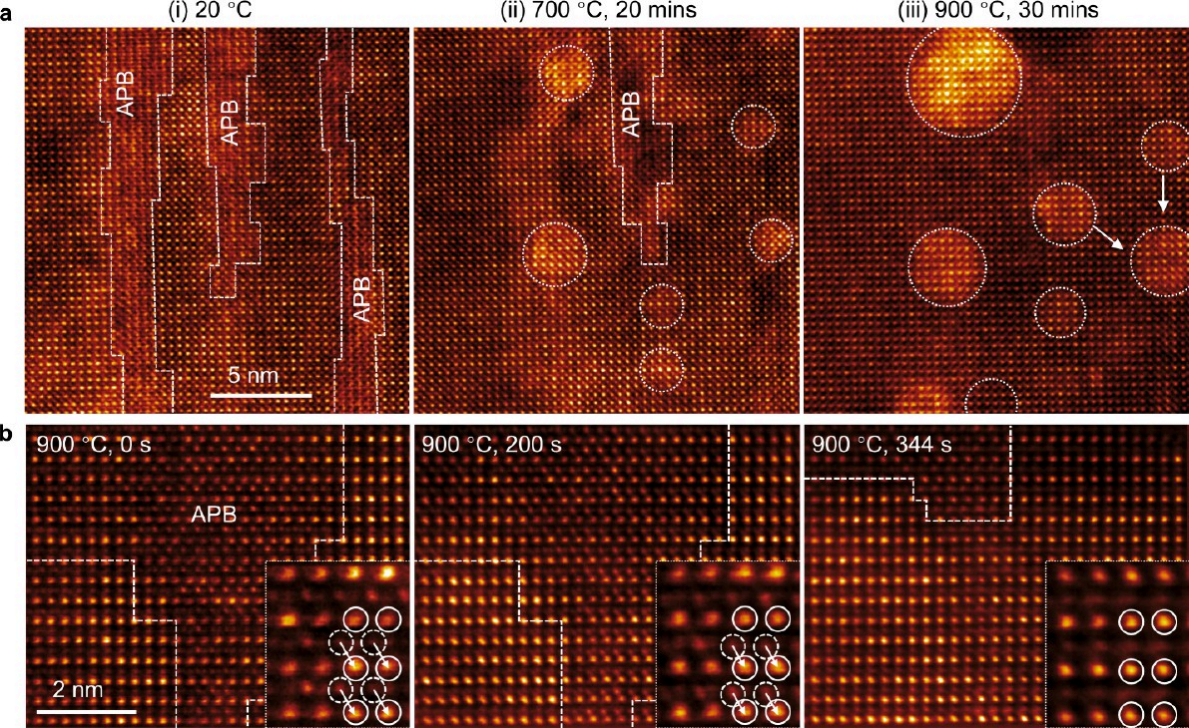
Disappearance of APBs under sequential annealing at different temperatures. (a) HAADF-STEM images illustrating the removal of APBs and ripening of NPs with increasing temperatures. (b) Sequential STEM images demonstrating the disappearance of APBs at 900 °C, viewed along the <110> zone axis. The insets illustrate the atomic displacement starting at 200 s, resulting in the elimination of the APB.

We have previously reported that the formation of APBs, known as fast diffusion pathways for Ni segregation and diffusion, is attributed to the nonstoichiometry with the presence of A-site deficiency and oxygen vacancies (V_O_) in the pristine film.^31^ It alters the oxygen coordination from corner-sharing to edge-sharing to accommodate V_O_, as schematically depicted in Figure 4a. The precise atomic structure of the APB, characterized by a translational vector of *t* = *a*/2 <011>, was confirmed by measuring the APB structures along two distinct zone axes (⟨010> (Figure 4b) and <110> (Figure 4c,d). Note that in Figure 4b,c, the APB is formed by an APND embedded within the matrix, resulting in the overlap of antiphase domains in the projection. Across this APB, the stacking sequence remains identical from the left to the right side. In contrast, the APB shown in Figure 4d separates two adjacent antiphase domains on the left and right, thereby providing a clearer representation of the intrinsic APB structure. Importantly, this difference does not indicate a structural variation between the two cases; rather, it arises from different sampling positions of the same type of APB during random TEM specimen preparation.

**Figure 4:**
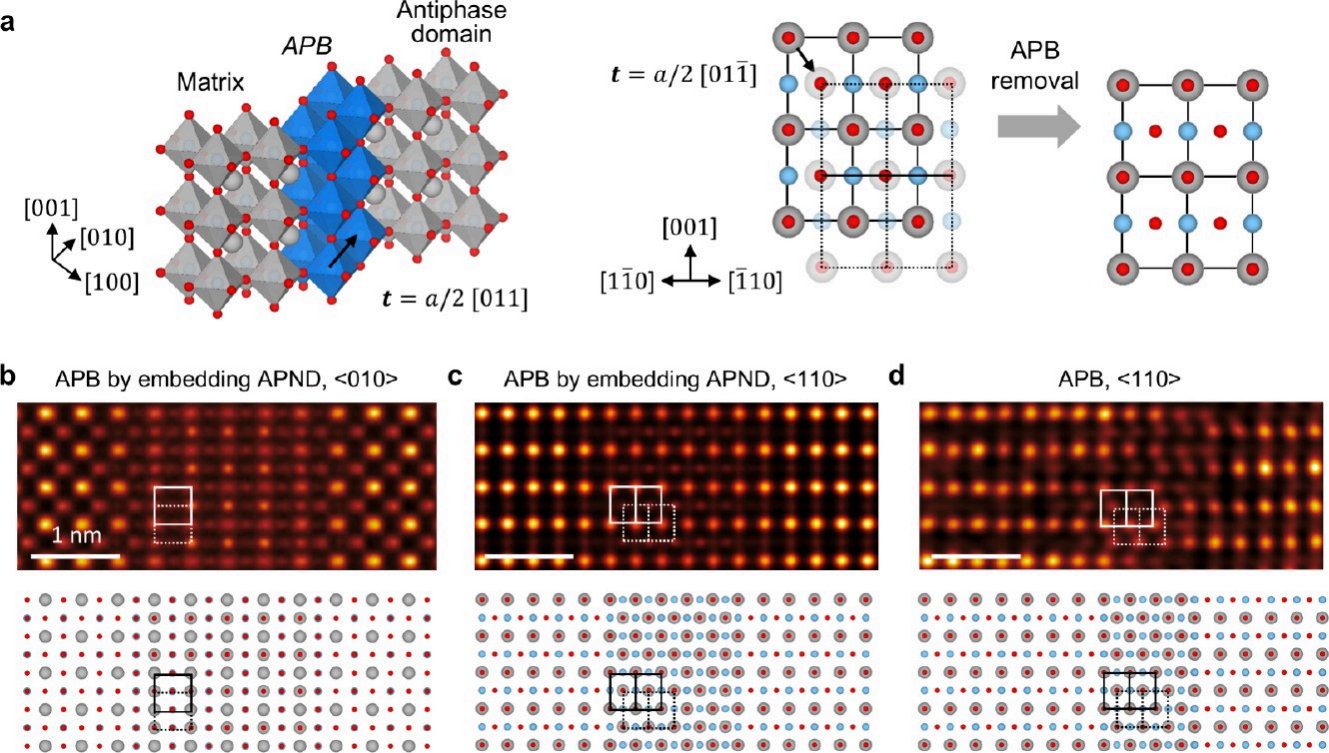
Atomic structure and translation of APBs. (a) Left: Atomic model of an APB formed by attaching a matrix and an antiphase domain with a translation vector, *t* = *a*/2 <011>. Right: Schematic diagram illustrating the atomistic mechanism of the APB removal process. (b, c) HAADF-STEM images of an APB generated by an APND embedded within the matrix, which leads to the overlap of antiphase domains in projection, viewed along the (b) <010> and (c) <110> zone axes. (d) HAADF-STEM image of an APB separating two adjacent antiphase domains, viewed along the <110> zone axis. The corresponding atomic structure models are shown beneath each image.

Regarding the gradual annihilation of APBs observed in our study, it proceeds via localized atomic rearrangements rather than a collective shift of entire domains. This process can be mediated by thermally activated migration of V_O_, which is known to be abundant at APBs and can facilitate local lattice reconstruction. Moreover, previous studies have reported that dislocation glide or climb can mediate the nucleation and annihilation of APBs in oxides.^35,36^ The movement of partial dislocations can locally shift the stacking sequence and eliminate the APB contrast without requiring long-range atomic motion. As seen in Figure 4d, the atomic arrangement across the APB reveals a consistent phase shift indicative of dislocation glide, supported by the preserved local order on both sides and the boundary’s propagation along a typical slip plane.

As exsolution progresses and NPs nucleate at the expense of B-site cations, ABO_3_ stoichiometry is restored as follows:^2,6^

$$A_{1-a}BO_{3-x}\to \left(1-a\right){\mathrm{ABO}}_{3-x\prime }+aB$$

As a result, the removal of APBs occurs concomitantly with the transition from edge-sharing back to corner-sharing due to V_O_ depletion induced by the exsolution of Ni cations. The EELS Ti L_2,3_ edge (Figure S3) displays two prominent peaks, L_3_ and L_2_, attributed to spin–orbit coupling in 3d orbitals. Crystal-field splitting further divides these peaks into t_2g_ and e_g_ sub-bands. In the bulk region, the valence state of Ti approximates 4+, resulting in distinct t_2g_ and e_g_ peaks in both the L_3_ and L_2_ edges (depicted in black). Conversely, at the APB, where edge-sharing oxygen coordination induces intrinsic oxygen deficiency,^31^ and V_O_ preferentially segregate,^37,38^ the Ti valence state is reduced from 4+ toward 3+, leading to a merging of the t_2g_ and e_g_ peaks in the L_2_ edge. This observation is consistent with the intensity reduction of the postpeak (particularly peak b) in the O K edge (Figure S3c), arising from the hybridization of the O 2p and Sr 4d orbitals. Following the removal of the APB, the discrepancies in Ti L_2,3_ and the O K edges between the bulk and the original APB region are substantially mitigated (Figures S3b,d).

We previously reported that stoichiometric films do not exhibit APBs, whereas nonstoichiometric films show pronounced APB formation in the as-grown state.^31^ This observation further supports the interpretation that APB annihilation during in situ reduction is associated with the transition from a nonstoichiometric to a stoichiometric state facilitated by the exsolution of excess Ni from the lattice.

Figure S4 further shows the coupled evolution of APBs and Ni NP exsolution with increasing temperature. Low-magnification HAADF-STEM images reveal that APBs progressively diminish as the temperature increases, accompanied by the emergence and growth of Ni NPs. Quantitative analysis demonstrates that the APB density decreases sharply at 700 °C, concurrent with a rapid increase in the number density of Ni NPs. This correlation indicates that Ni exsolution drives the transition from an initially nonstoichiometric lattice toward a stoichiometric state, leading to APB annihilation. Upon further increasing the temperature above 900 °C, the remaining APBs continue to decrease, while the NP number density decreasess and the average NP radius increases. This result suggests coarsening and partial sublimation of Ni at elevated temperatures.

Notably, reduction of LSNT thin films grown on (LaAlO_3_)_0.3_(Sr_2_AlTaO_6_)_0.7_ (LSAT) (001) with a misfit strain of −1.5% produces a similar result (Figure S5), suggesting that this mechanism is universal and independent of the lattice strain. We note that the epitaxial interface exhibits no discernible changes before and after exsolution. The interface remains sharp, with no contrast variation in the STEM images, indicating that the interfacial structure and crystallinity are preserved throughout the reduction process.

### Particle Anchoring Mechanism at Elevated Temperatures

We next focused on the film surface to directly investigate the high-temperature (1000 °C) dynamics of exsolved NPs and the socketing process using in situ STEM (Figure 5). The exsolved NP with a diameter (d) larger than ∼4 nm shows lateral migration and rotation on the surface prior to anchoring (Figure 5a–c). During this process, the particle continuously adjusts its crystallographic orientation while migrating along the surface until epitaxial alignment with the perovskite lattice is established. Once alignment between the Ni <100> facets and the <100> facets of the perovskite support is achieved, reactive wetting is initiated, leading to progressive embedding of 1–2 unit cells and the formation of a stable socketed interface. The establishment of this low-energy epitaxial interface ultimately results in firm anchoring of the particle.

**Figure 5:**
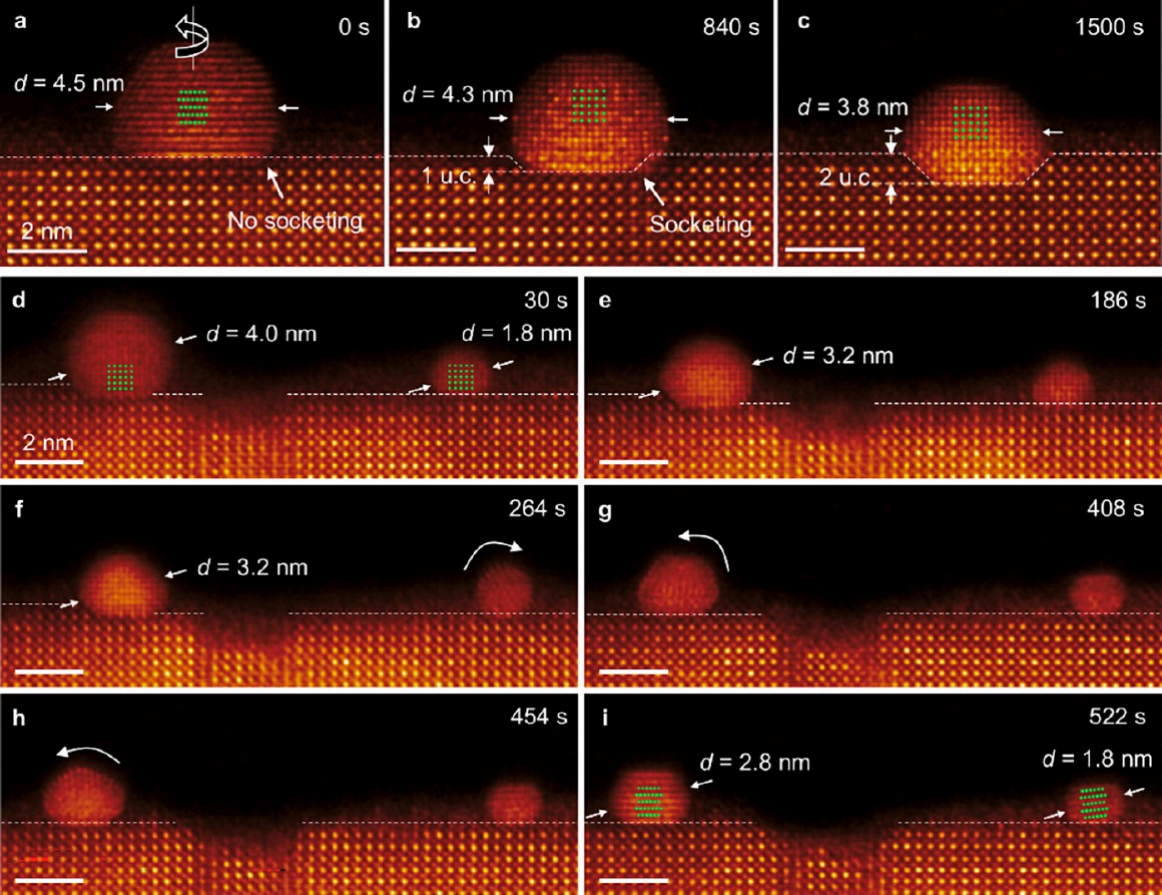
Thermally activated rotational and translational reconfiguration followed by reactive wetting of NP. (a–c) Self-reconfiguration and subsequent reactive wetting of NP. (d–i) Time sequence of HAADF-STEM images, illustrating the rotation and migration of NP. The in situ imaging was done at 1000 °C.

In contrast, NPs approaching a diameter of approximately 2–3 nm lose stable socketing and crystallographic alignment. As illustrated in Figure 5d–i, these smaller particles display enhanced rotational and lateral mobility, accompanied by continuous size reduction due to the accelerated sublimation of Ni species at elevated temperatures. The shrinkage progressively destabilizes the interfacial alignment, thereby preventing the formation of a stable wetting configuration.

Therefore, based on these observations, we identify two key findings regarding the anchoring mechanism of exsolved NPs. First, a critical particle size above ∼3 nm is required to trigger reactive wetting and, thereby, enable stable anchoring. Second, NPs undergo dynamic migration and rotation prior to anchoring, indicating that crystallographic faceting with the perovskite lattice by reactive wetting is a prerequisite for subsequent stabilization.

### Surface Pit Formation during High-Temperature Reduction

The continuous chemical state change from prolonged reduction induces volatilization of the surface layers, as depicted in Figure S6. The formation of the pit on the surface starts at 700 °C within ∼20 min, and the depth of the pit further increases with raising the temperature, indicating the sublimation of the surface layer during the reduction process. This observation is consistent with the pit formation observed in the AFM measurements of the ex situ reduced films (Figure 1b,c). Figure S7 and Movie S3 further reveal the pit formation at 800 °C.

We further performed low-magnification in situ STEM imaging to capture the global evolution of both the surface and interior regions during high-temperature reduction (Figure 6). Dashed circles in Figure 6a highlight the positions of exsolved Ni NPs after reduction at 1000 °C. As indicated by triangles, surface pits form and extend from the surface into the film interior and are decorated with NPs, suggesting that they coincide with the locations of former APBs. A pronounced decrease in both the size and number density of NPs is observed, particularly along the pit lines, indicating progressive sublimation of Ni NPs.

**Figure 6:**
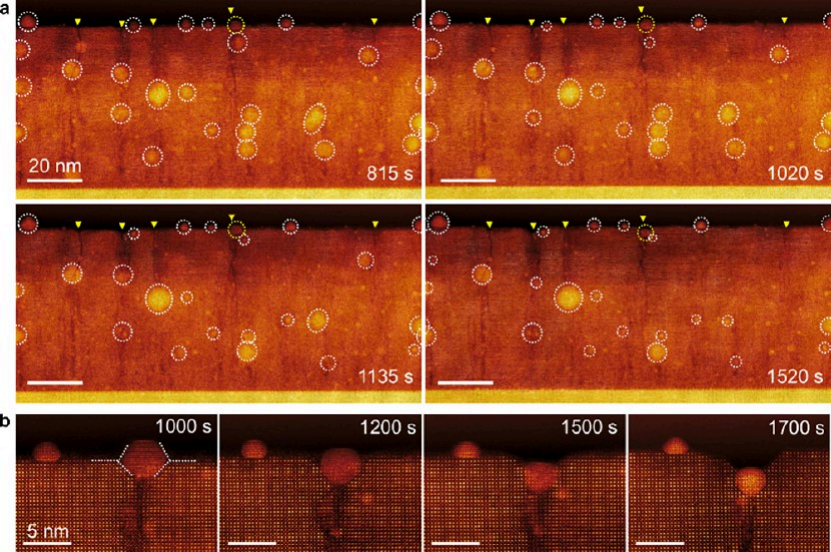
Global evolution of Ni NPs and surface pit formation. (a) Low-magnification HAADF-STEM images, illustrating the simultaneous reduction in NP size and density and the development of surface pits. (b) Sequential images capturing the sliding of a single NP into an expanding pit during high-temperature annealing at 1000 °C.

Figure 6b shows two NPs within the field-of-view, with the larger NP initially positioned atop an expanding surface pit. As the pit deepens, the NP slides into the widening opening and gradually reorients, ultimately adopting a preferred crystallographic orientation alignment with the <010> zone axis parallel to the viewing direction. Notably, the NP shrinks more rapidly as it becomes increasingly embedded beneath the oxide surface, eventually reaching a diameter of ∼3 nm.

These low-magnification observations clarify the distinct behaviors of the surface and interior NPs. Particles on the surface exhibit pronounced lateral mobility and agglomeration due to enhanced surface diffusion. In contrast, NPs formed within the bulk lattice exhibit limited migration due to confinement within the lattice. Note that the long-range migration of some NPs within the interior region is likely related to their proximity to the exposed side surfaces of the TEM specimen, where the presence of side surfaces can promote enhanced mobility and coalescence as opposed to those embedded within the bulk.

### Secondary Phase Formation during High-Temperature Reduction

As shown in Figure S8, a mound-shaped particle is formed on the surface when reducing at 900 °C within 2 h. This indicates the formation of a secondary phase on the surface due to chemical state changes and the sublimation of exsolved Ni NPs during the prolonged reduction. It is observed that the secondary phase grows at step ledges rather than on the flat surface, which was further confirmed at 1000 °C (Figure 6, Figure S9, and Movie S4). Combining crystal structure and elemental composition analyses (Figure 7c and Figure S10), the secondary phase is identified as La_2_TiO_5_, which has been observed in a bulk polycrystalline sample.^27^ Figure 6d illustrates the details of the secondary phase growth process. The initial image displays a clean step without La_2_TiO_5_ formation. During the sublimation by ledge migration of surface steps, indicated by triangles, atomic layers with lower intensity are stacking up, indicating substantially heavier elements of the host lattice are sublimated preferentially. EDS maps represent that the dominant sublimated elements are Sr and Ni (Figure 7c and Figure S11).^39^ The small Ni NPs are likely embedded within the La_2_TiO_5_ islands (Figure S12), and even at the twin boundaries (TB) of La_2_TiO_5_ (Figure 7c).

**Figure 7:**
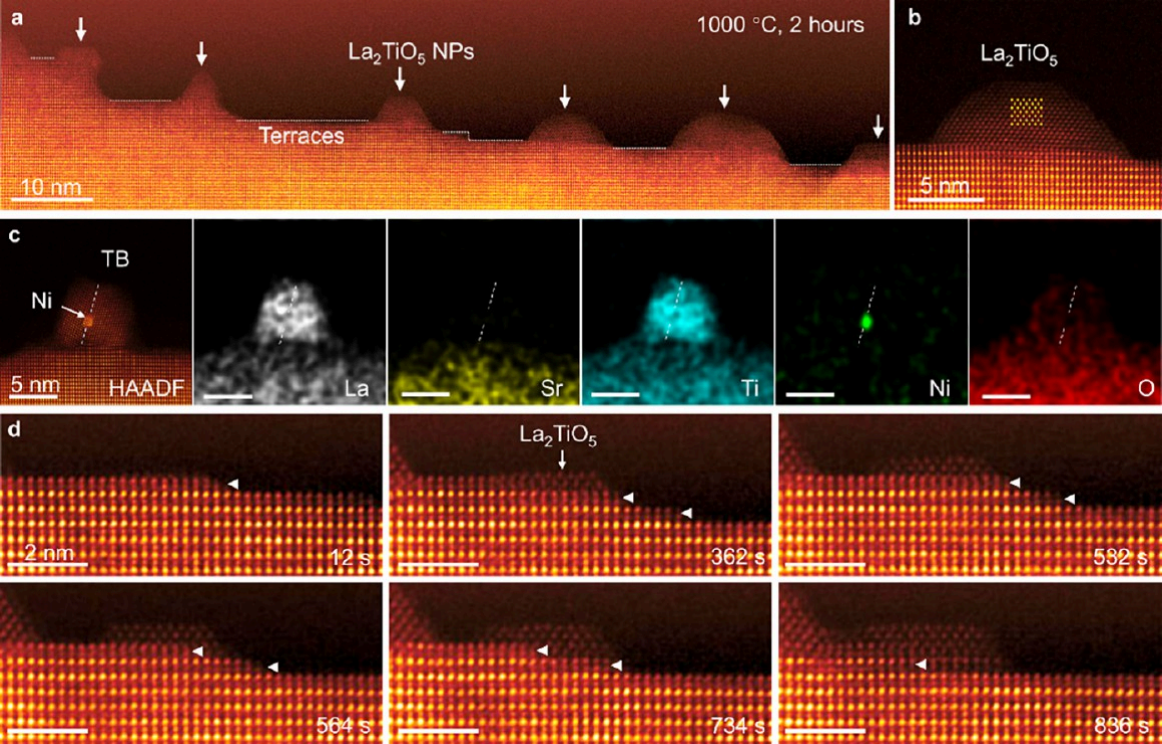
Formation of the secondary phase, La_2_TiO_5_. (a) low-magnification HAADF-STEM image after annealing at 1000 °C for 2 h, illustrating the formation of the secondary phase. (b) Magnified image revealing the atomic structure of the secondary phase. (c) HAADF-STEM image and corresponding EDS maps displaying the composition of the secondary phase and Ni NP encapsulation at the twin boundary. (d) Sequential images showing the formation of secondary phase through surface ledge migration.

### Sequential Chemical Changes Revealed by In Situ EELS

In situ EELS was performed to investigate the chemical state changes of the film lattice during the exsolution process (Figure S13). The Ti L_2,3_ edge spectra (Figure S13b) show two characteristic doublets corresponding to the crystal-field splitting of the Ti 3d orbitals into t_2g_ and e_g_ states. As the reduction proceeds, the characteristic Ti^3+^ components, appearing near ∼461 (L_3_ edge) and ∼466 eV (L_2_ edge), gradually increase in intensity. This Ti reduction arises from the exsolution of Ni cations from the perovskite lattice, which leaves behind a La-doped SrTiO_3_-like host lattice with an increased electron occupancy in the Ti 3d orbitals. This temporal change in the lattice results in the disappearance of APBs.

In addition, the generation of V_O_ during reduction further enhances the partial reduction of Ti, as corroborated by the polarized O K edge spectra. The O K edge spectra (Figure S13c) exhibit three main features labeled a, b, and c, associated with the O 2p-Ti 3d (t_2g_), the O 2p-Sr 4d, and the O 2p-Ti 4sp hybridized states, respectively. With increasing reduction temperature, particularly above 900 °C, the intensity of the b peak (∼537 eV) markedly decreases, indicating a significant reduction in the oxygen valence state and a suppression of O 2p-Sr 4d hybridization. This suppression of the “b” peak reflects the decreased number of unoccupied O 2p–Sr 4d hybridized states, likely associated with changes in the Sr–O coordination environment due to partial Sr sublimation. These changes confirm that high-temperature reduction (∼900 °C) drives a pronounced electronic reconstruction of the perovskite lattice, characterized by oxygen deficiency and reduced Ti valence, which ultimately drives perovskite lattice decomposition in conjunction with the formation of secondary phases.

## Conclusions

In this study, we have investigated the overall phase evolution of NP exsolution in epitaxial perovskite thin films through combined in situ transmission electron microscopy and ex situ structural and surface characterization. The atomic-scale transformation and degradation processes, including the chemical evolution of the perovskite lattice and the resulting changes in the NP behavior, are illustrated in Figure 8. In the pristine state, the nonstoichiometric lattice contains oxygen-deficient APBs that serve as fast diffusion channels for Ni, enabling NPs to nucleate and migrate toward the surface during reduction. With further progress of reduction, the lattice transiently approaches a stoichiometric state during which the APBs remarkably disappear.

**Figure 8:**
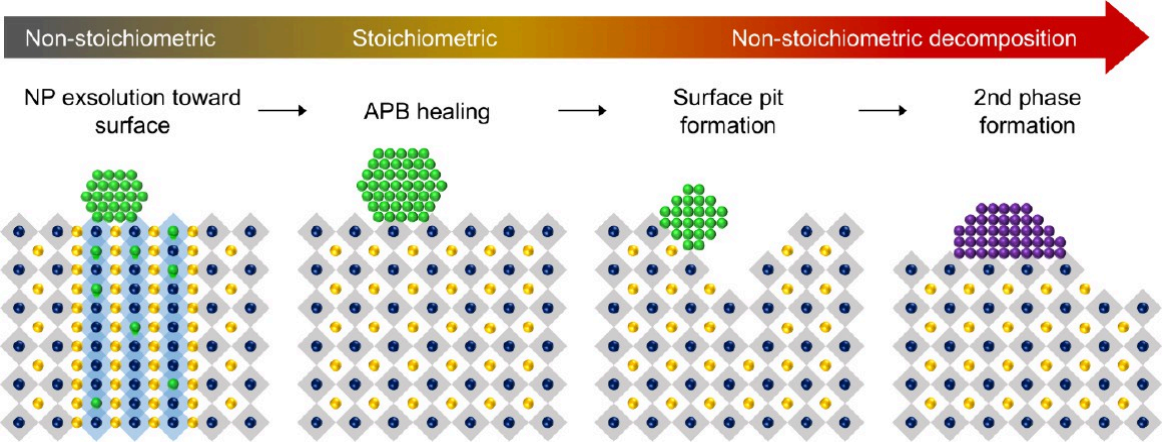
Schematic illustration of the atomic-scale transformation process of the exsolved nanoparticle in the thin film via reduction. The chemical transformation of the perovskite lattice is illustrated by the arrows in the upper part of the figure. The yellow, blue, and green spheres denote La/Sr (A-site), Ti (B-site), and Ni (B-site) ions, respectively. The blue and gray polyhedral represent the octahedra at the APBs and perovskite lattice, respectively. The green and purple ions on the surface indicate the Ni NP and secondary phase (La_2_TiO_5_), respectively.

Moreover, continued high-temperature reduction induces surface pit formation followed by the emergence of a La_2_TiO_5_ secondary phase, indicating a reversion of the lattice toward a nonstoichiometric state at the surface. While pure SrTiO_3_ is generally regarded as structurally stable under high-temperature annealing, where reduction primarily generates oxygen vacancies, our system differs fundamentally. The present experiments were performed under strongly reducing conditions using initially nonstoichiometric films containing a high density of APBs and volatile cations, including excess Ni and Sr. Thus, under these conditions, prolonged annealing promotes preferential volatilization of Ni and Sr, which are more volatile than La and Ti.^40–45^ This selective cation loss, together with oxygen vacancy formation, drives substantial compositional reconstruction of the lattice, manifested by surface pit formation and subsequent La_2_TiO_5_ phase development via surface ledge migration. Namely, these transformations are not characteristic of SrTiO_3_ base material under classical reduction conditions but instead result from the combined effects of initial nonstoichiometry, defect-assisted diffusion, and preferential cation volatilization.

Thus, these results establish an atomic-scale understanding of NP exsolution in perovskite thin films, spanning defect-assisted nucleation, defect annihilation, and ultimately high-temperature degradation and secondary phase formation. The discovery that exsolution itself removes APBs reveals an unexpected feedback mechanism between the lattice stoichiometry and defect chemistry. Conversely, the recovery of nonstoichiometry and the subsequent decomposition of both the lattice and the NPs uncover a universal degradation pathway that needs to be considered for applications. These insights not only challenge the prevailing assumption of long-term NP stability in exsolved systems but also provide new guidelines for designing durable exsolution-based catalysts and energy materials.

## Methods

### Thin Film Preparation

The 60 nm-thick nonstoichiometric thin films were grown on LSAT (001) and GSO (110) substrates using nonstoichiometric La_0.2_Sr_0.7_Ni_0.1_Ti_0.9_O_3‑δ_ (LSNT) target having 10% A-site deficiency by pulsed laser deposition. The O partial pressure, deposition temperature, laser fluence, and repetition rate were fixed at 20 mTorr, 700 °C, 1 J cm^–2^, and 5 Hz, respectively. For the ex situ XRD and AFM measurements, the reduction of the grown thin films was performed in a tube furnace with a continuous flow of gas mixture of Ar (95%) + H_2_ (5%) at 850 and 950 °C with the heating and cooling rates of 5 °C·min^–1^.

### Film Characterizations

The theta–2theta scan was performed using a high-resolution X-ray diffractometer (D8 Discover, Bruker) under Cu Kα radiation operated at 40 kV and 40 mA. The surface topography was measured by AFM (Dimension Icon, Bruker) with ScanAsyst mode. XPS measurements were performed using a Thermo Fisher K-alpha Plus system after mild Ar ion sputtering of the sample surface to remove surface contamination.

### TEM Sample Preparation

Cross-sectional TEM specimens were prepared by using focused ion beam (FIB) milling. The prepared lamella by FIB was attached to a Protochips heating chip for in situ heating experiments. At the final stage of FIB milling, a low-energy Ga^+^ ion beam at 2 kV was used to reduce beam damage.

### Scanning Transmission Electron Microscopy

The in situ heating experiments were conducted in the vacuum environment (∼10^–7^ Torr) of a field-emission Cs-corrected scanning transmission electron microscope (S/TEM), specifically a SpectraUltra model by ThermoFisher, operating at 300 kV. A Protochips heating holder facilitated controlled in situ heating.

Atomic-scale characterization during the in situ heating experiment was recorded in annular dark-field (ABF) and high-angle annular dark-field (HAADF) imaging modes. The imaging modes were set with detector angle ranges of 7.5 to 17 mrad for ABF and 52 to 200 mrad for HAADF. The convergence semiangle for forming the focused probe was maintained at 21.5 mrad. To ensure minimal electron beam damage, the electron beam current was optimized by balancing the image contrast. The electron dose rate was maintained at approximately 10^6^ electrons per nm^2^ per second for all in situ imaging experiments. The statistical noise floor in all STEM images was removed using local 2D Wiener filtering implemented using commercial software (HRTEM Filter Pro, HREM Research Ltd.).

Energy-dispersive X-ray spectroscopy (EDS) was acquired under a 30 mrad convergence angle using a spectrometer with a detectable solid-angle of 4.04 srad in STEM imaging mode. EDS maps, comprising approximately 1000 frames at a speed of 0.5 s per frame, were acquired. Specimen drift correction was implemented automatically during acquisition in Velox software. EDS maps underwent processing by using an average filter to minimize random noise without introducing artifacts.

Electron energy-loss spectroscopy (EELS) line scans were recorded with energy ranges of 400 to 600 eV (for the O K edge and Ti L_2,3_ edges) using an EEL spectrometer (Gatan GIF Quantum ER, USA) with an energy resolution of 0.6 eV. The energy dispersion and dwell time per pixel are 0.1 eV and 0.5 s, respectively. The loss energy of the core-loss EELS data was calibrated by tracking the energy drift of the zero-loss peak, which was simultaneously recorded with the core-loss data. The electron dose rate is about 10^6^ e^–^·nm^–2^·s^–1^.

## Supplementary Material











## Notes

(Supporting Movie S1) In situ STEM movie showing the APB depletion (MP4)
(Supporting Movie S2) Magnified in situ STEM movie showing the APB depletion (MP4)
(Supporting Movie S3) Magnified in situ STEM movie showing the surface pit formation during high-temperature annealing (MP4)
(Supporting Movie S4) In situ STEM movie showing the formation of secondary phase by surface ledge migration at high temperature (MP4)
AFM, XPS, TEM, EDS maps, and EELS spectra of pristine and exsolved thin films (Figures S1–S13) (PDF)
