# Supplementary material for: Atomic-Scale Degradation Mechanisms during Nanoparticle Exsolution in Thin Films
Source: ACS Nano. 2026 Apr 15;20(16):12296–305. doi: 10.1021/acsnano.5c20606 (PMC13131044; doi:10.1021/acsnano.5c20606)
Supplement: Supplementary file 5 [file nn5c20606_si_005.pdf]

Supporting Information

Atomic-Scale Degradation Mechanisms during Nanoparticle Exsolution in Thin Films

Yaolong Xing[†‡]*, Hyojin Yoon[§‖], Haeseong Jeong[§‖], Dongchang Kim[⊥], Stuart S. P. Parkin[§‖⊥], Hyeon Han[§‖⊥]*, Sang Ho Oh[†#]*

[†]Institute for Energy Materials and Devices, Korea Institute of Energy Technology (KENTECH), Naju 58330, Republic of Korea.

[‡]Department Structure and Nano-/Micromechanics of Materials, Max-Planck-Institute for Sustainable Materials, Dusseldorf 40237, Germany.

[§]Department of Materials Science and Engineering, Pohang University of Science and Technology (POSTECH), Pohang 37673, Republic of Korea.

[‖]Korea-Max Planck-PSI Center for Quantum Emergent Spintronics (KOMQUEST), Pohang 37673, Republic of Korea.

[⊥]Max Planck Institute of Microstructure Physics, Weinberg 2, Halle (Saale) 06120, Germany.

[#]Department of Energy Engineering, Korea Institute of Energy Technology (KENTECH), Naju 58330, Republic of Korea.

*Corresponding authors:

Yaolong Xing: y.xing@mpi-susmat.de

Hyeon Han: hyeonhan@postech.ac.kr

Sang Ho Oh: shoh@kentech.ac.kr

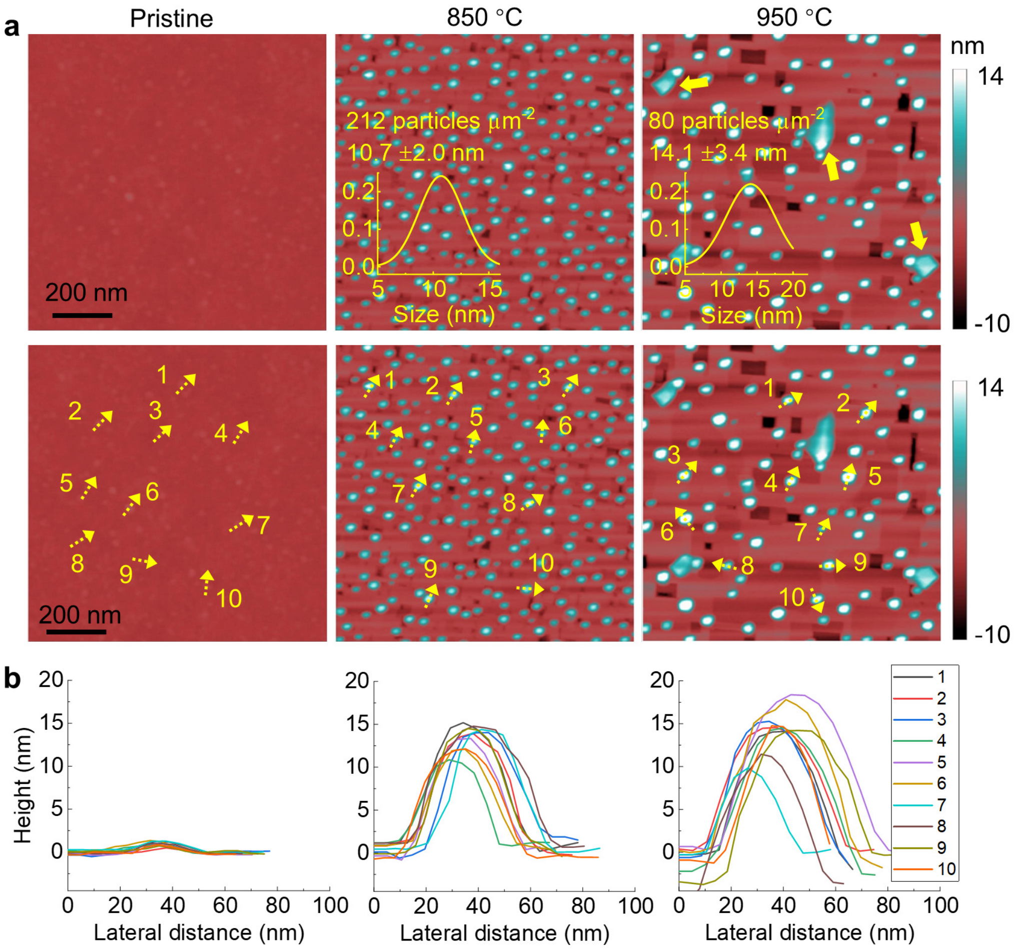

Figure S1. Surface particle analysis of the films before and after reduction. a, AFM images and **b**, particle heights for pristine, 850 °C and 950 °C reduced films. The insets in the upper panels indicate the population density, average particle size (height), and particle size distribution curve for the reduced films. A significant increase in particle height (size) is shown after the reduction. The surface roughness (root mean square; rms) is 0.22 nm, 3.15 nm, and 3.96 nm for pristine, 850 °C and 950 °C reduced films, respectively.

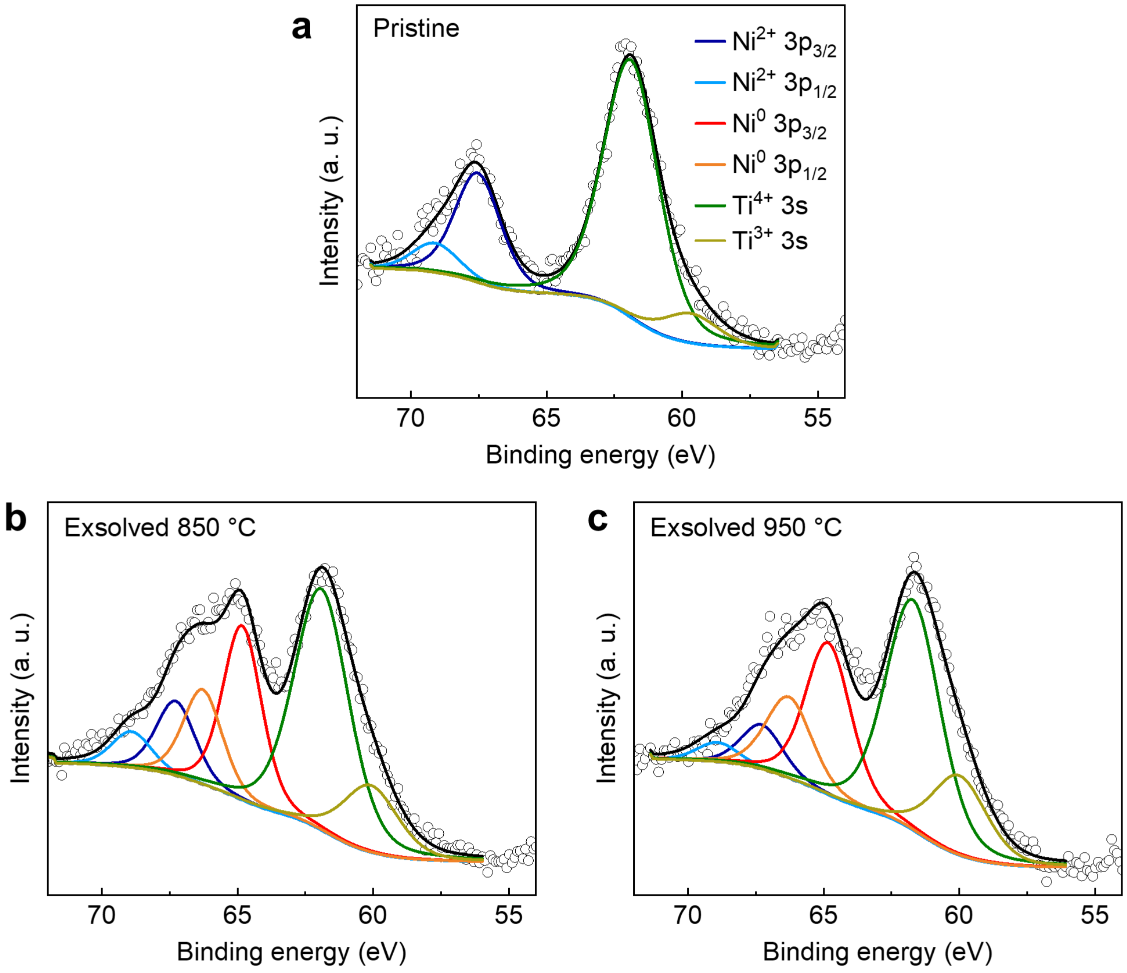

Figure S2. X-ray photoelectron spectroscopy (XPS) spectra of pristine and exsolved thin films. **a**, Pristine thin film, **b**, thin film exsolved at 850 °C, and **c**, thin film exsolved at 950 °C. Quantitative analysis of the XPS peak area ratios indicates that metallic Ni^0 accounts for ~70% of the total Ni content in the 850 °C reduced film and about 80% in the 950 °C reduced film. Furthermore, Ti^{3+} fraction increases from about 10% in the pristine film to 22% and 26% in the films reduced at 850 °C and 950 °C, respectively, indicating gradual reduction of Ti with increasing exsolution.

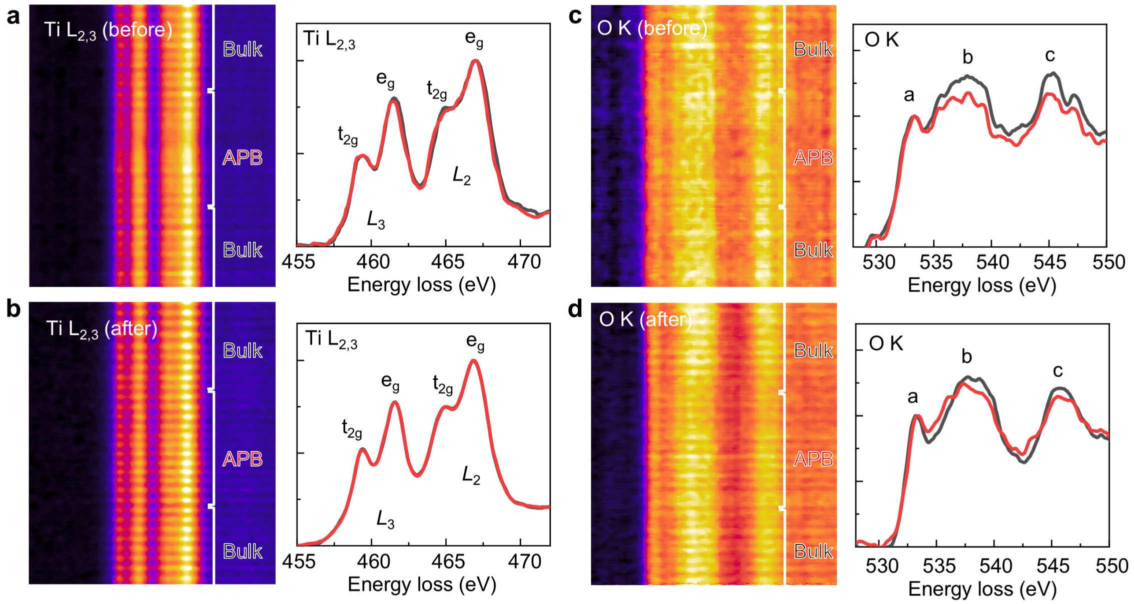

Figure S3. EELS measurements before and after APBs disappearance. a, line scan EELS Ti $L_{2,3}$ edge across APB. Left panel, spectrum image. Right panel, averaged spectrum of bulk region (black) and APB region (red). **b**, as of **a**, but after APB removal. **c**, line scan EELS O K edge across APB. Left panel, spectrum image. Right panel, averaged spectrum of bulk region (black) and APB region (red). **d**, as of **c,** but after APB removal.

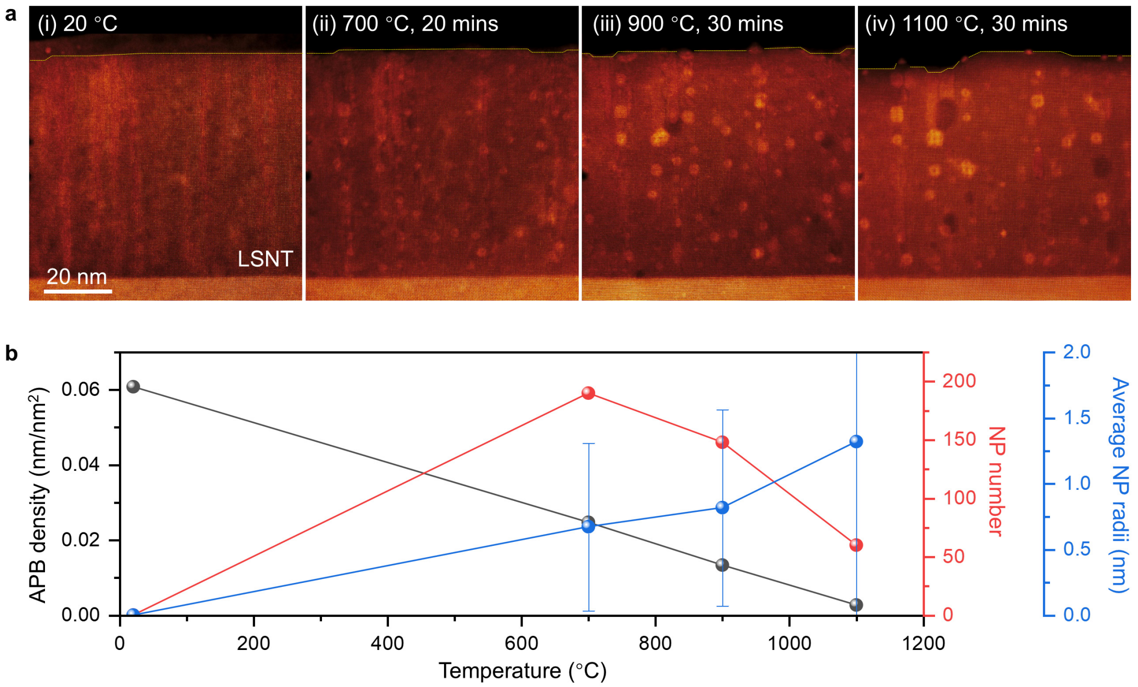

Figure S4. Coupled evolution of APBs and NPs with increasing temperature. a, Low-magnification HAADF-STEM images illustrating the exsolution process under various temperatures. APBs are disappeared as increasing temperature. **b**, Quantitative evolution of the APB density (defined as the average total vertical length per projected area), together with the number density and average radius of NPs within the same area.

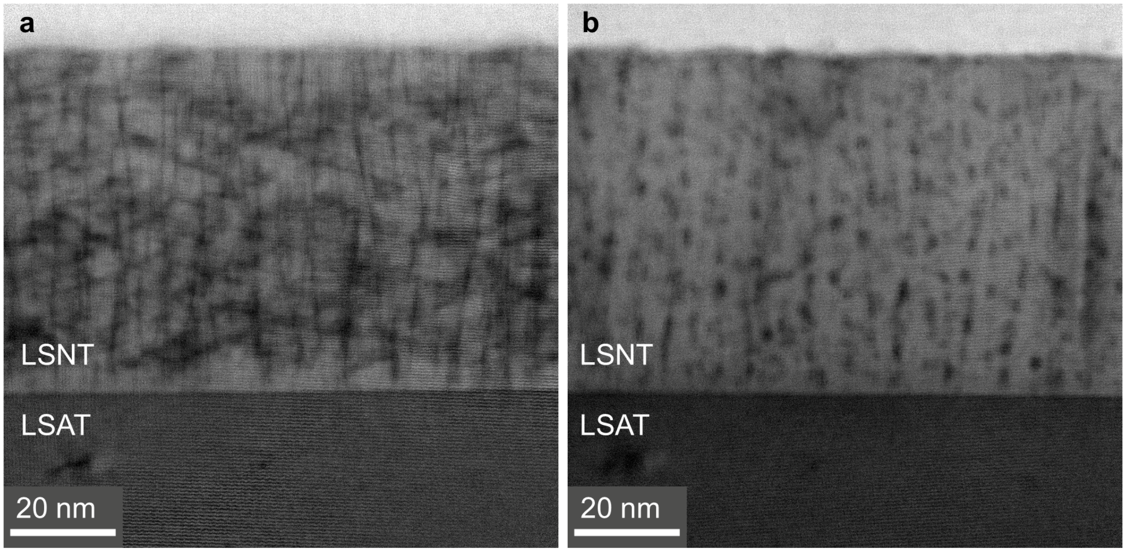

Figure S5. Disappearance of APBs in LSNT/LSAT thin film through exsolution. a, ABF-STEM image of the pristine LSNT/LSAT thin film. Vertically aligned dark lines represent APBs. **b**, ABF-STEM image after heating at 700 °C for 30 minutes. The disappearance of vertically aligned lines indicates the depletion of APBs. Instead, the formation of dark spots indicates the generation of NPs through exsolution.

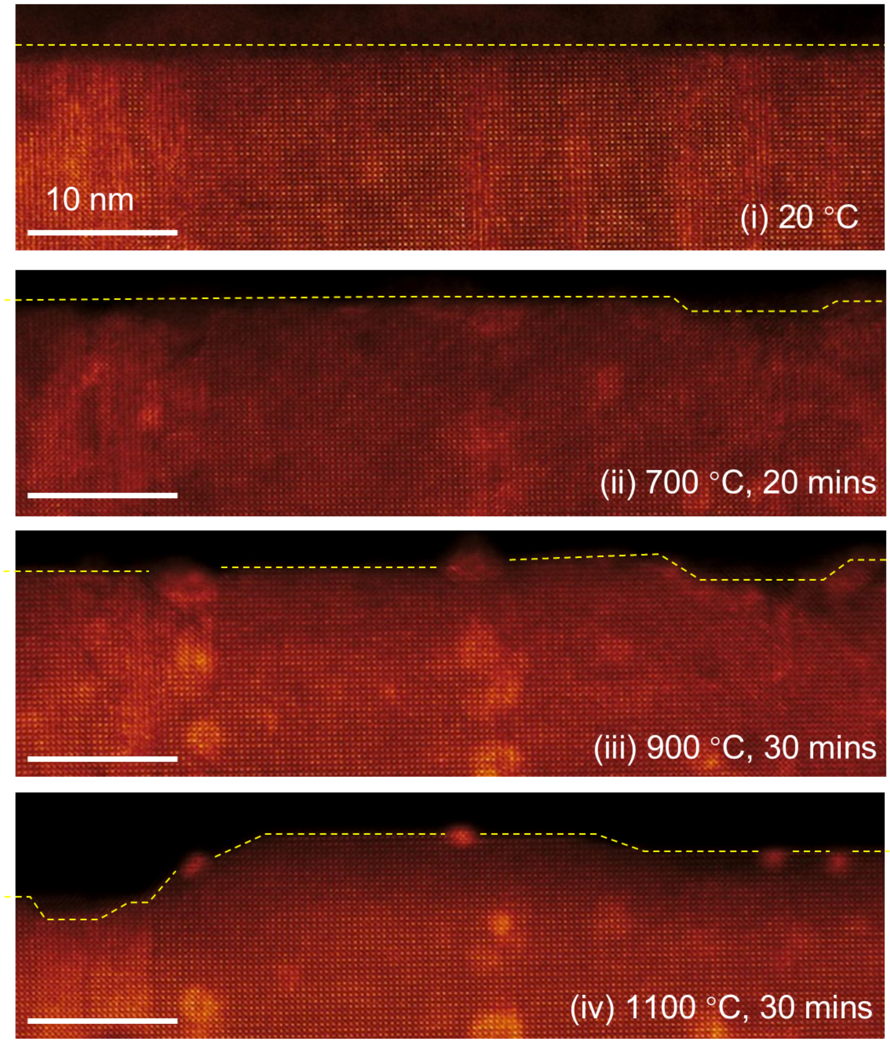

Figure S6. The formation of the surface pit. The evolution of surface morphology as increasing the temperature. The pit is formed on the surface after reduction at 700 °C, and the pit depth is increasing with raising the temperature. The surface nanoparticle size also decreases with increasing temperature.

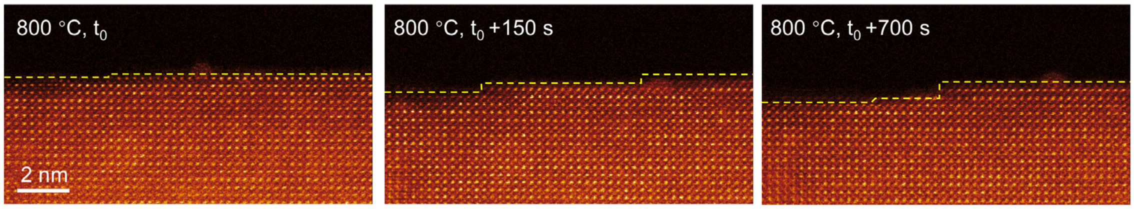

Figure S7. Surface pit formation at 800 °C. ABF-STEM images during the reduction at 800 °C, showing the formation of the surface pit.

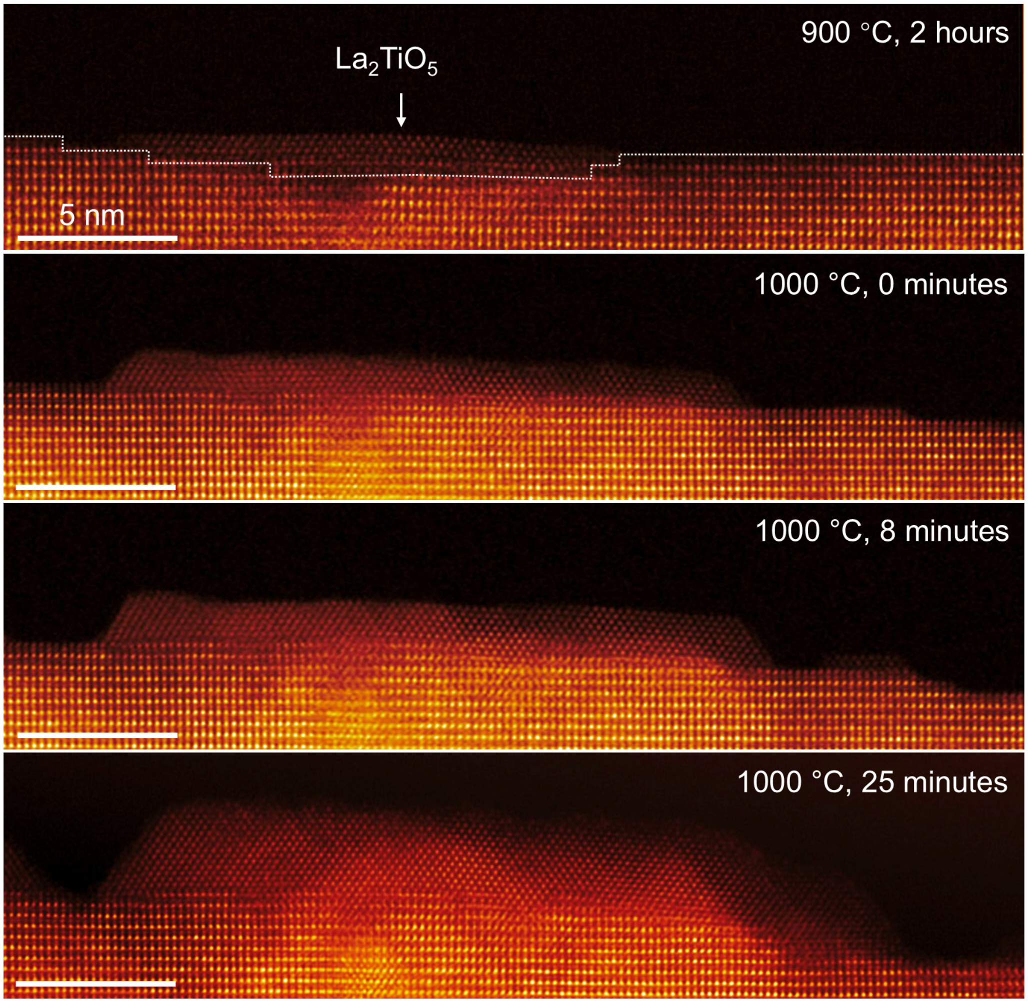

Figure S8. The formation of the second phase by surface ledge migration. Time series HAADF-STEM images illustrating the formation of second phase by surface ledge migration above 900 °C.

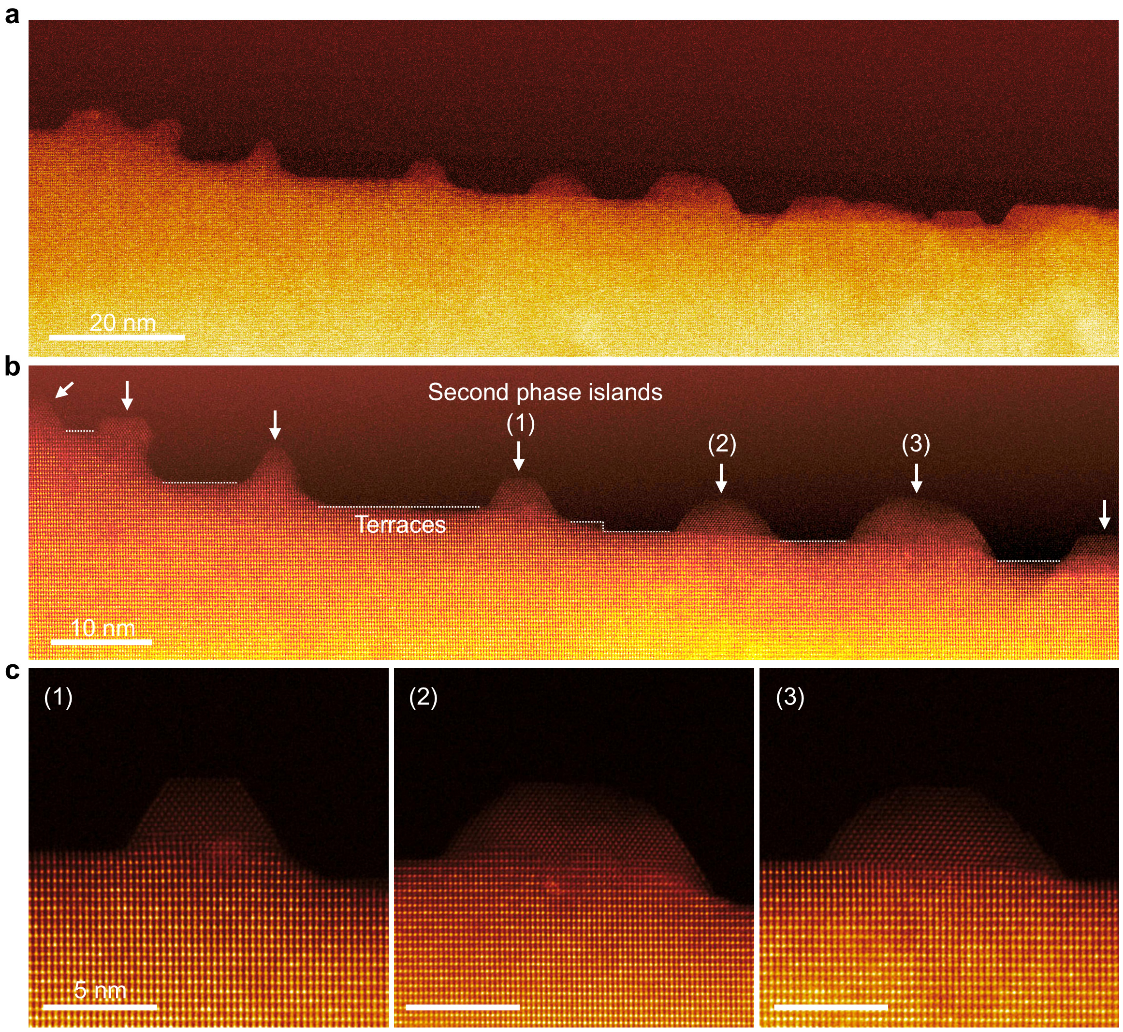

Figure S9. The formation of the secondary phase following high-temperature annealing. **a**, low-magnification HAADF-STEM image illustrating the sample morphology with the appearance of island-like secondary phase on the surface. The image was captured after 2 hours of annealing at 1000°C. **b**, higher magnification HAADF-STEM image showing a closer view of the secondary phase islands. **c**, atom-resolved image of an individual island, revealing the atomic arrangement of the secondary phase.

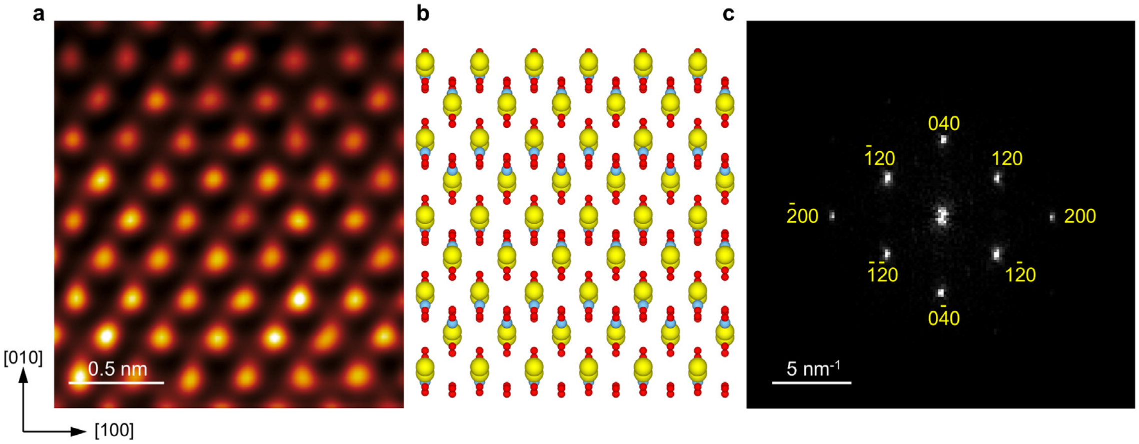

Figure S10. Crystal structure of secondary phase La_2TiO_5. a, HAADF image of La_2TiO_5. **b**, crystal structure of La_2TiO_5 viewing along [001] zone axis. **c**, FFT of HAADF image.

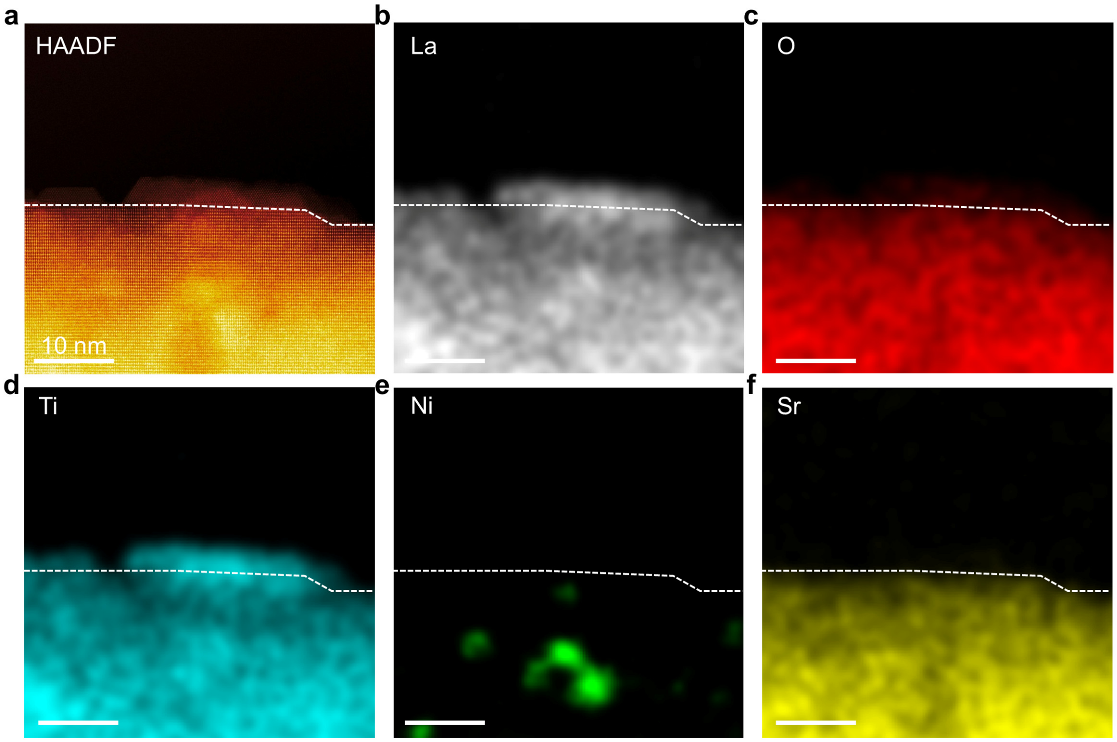

Figure S11. Composition of the secondary phase island in Figure 6. a, HAADF-STEM image. **b-f**, EDS maps of **b**, La, **c**, O, **d**, Ti, **e**, Ni and **f**, Sr.

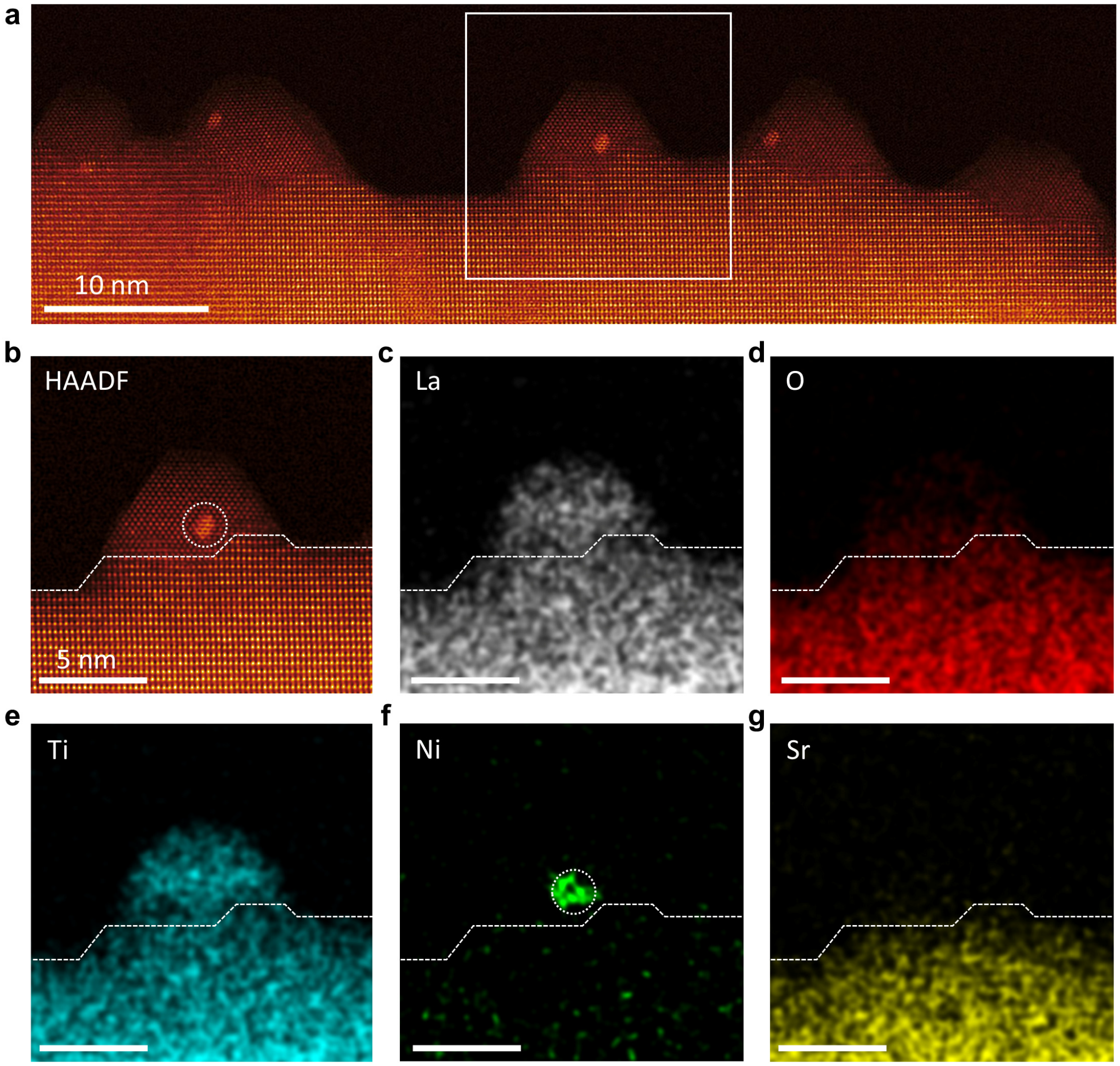

Figure S12. Secondary phase encapsulating Ni NPs. a, low magnification HAADF-STEM image showing Ni NPs are encapsulated by secondary phase islands. **c-g**, EDS maps of **c**, La, **d**, O, **e**, Ti, **f**, Ni and **g**, Sr.

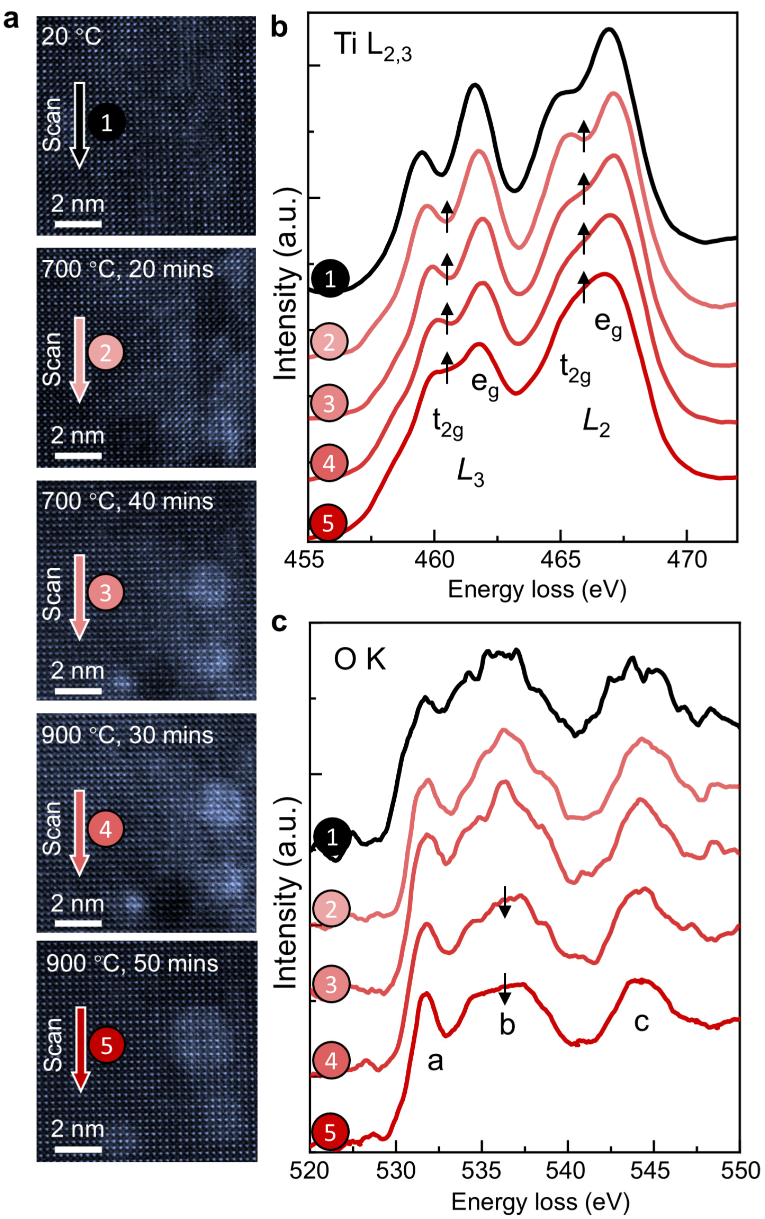

Figure S13. EELS measurement during in-situ heating. a, HAADF-STEM images captured during EELS acquisition. **b**, EELS spectrum of the Ti $L_{2,3}$ edge. **c**, EELS spectrum of the O K edge.

Supporting Movie Captions

Movie S1. In-situ STEM movie showing the APB depletion. The measurement was done at 900 °C. The playback rate is 30 times fast.

Movie S2. Magnified in-situ STEM movie showing the APB depletion. The measurement was done at 900 °C. The playback rate is 30 times fast.

Movie S3. Magnified in-situ STEM movie showing the surface pit formation during high temperature annealing. The measurement was done at 800 °C. The playback rate is 10 times fast.

Movie S4. In-situ STEM movie showing the formation of secondary phase by surface ledge migration at high temperature. The measurement was done at 1000 °C. The playback rate is 50 times fast.
